# Competing perspectives on energy transitions: a global comparison

**DOI:** 10.1007/s41358-020-00214-7

**Published:** 2020-04-07

**Authors:** Miranda A. Schreurs

**Affiliations:** Environment and Climate Policy, Technical University of Munich, Richard-Wagner-Straße 1, 80333 Munich, Germany

## Introduction

There are two competing narratives regarding Germany’s energy transition. The one is that Germany should pioneer the path to a carbon-neutral energy future in response to rapidly rising greenhouse gas emissions and pursuant climate change. The other is that the German energy transition is misguided and could harm the competitiveness of German industries. Those embracing this view vary in the extent to which they accept the threat of climate change as real and urgent. Thus, some would argue to abandon the energy transition altogether and others to slow its pace. This article examines these two narratives putting the German case in international comparison. It does so looking at the situation in the United States, the European Union, China, and Japan.

## The stickiness of dominant narratives and path dependencies

Political, economic, and social decisions tend to follow dominant narratives. Narratives create realities. They are embedded with ideas which influence behavior and shape cultures and which become embedded in institutions. Narratives influence what we see and think about, how we see things, and how we behave. They create cultural, institutional, and infrastructural path dependencies.

For the last century, the dominant narrative has been that growth requires access to cheap energy and natural resources. Cheap energy has been defined as fossil fuels and nuclear energy. The industrial revolution spurred a massive growth in the demand for fossil fuels while also increasing capabilities to extract fossil fuels and mineral resources from previously unimaginable depths and locations. Coal, oil, and gas and later nuclear energy fueled the economic growth that brought wealth to Europe and North America and more recently to China and East Asia. For many decades coal miners were organized in labor unions that defined regional and at times national politics. Oil and gas companies (Sinopec, Royal Dutch Shell, China National Petroleum, Saudi Aramco, BP, Exxon Mobil …) continue to be among the wealthiest companies in the world. Fossil fuel wealth has also helped to keep a narrative alive that fossil fuel and nuclear energy is essential for the well-being of modern societies, that fossil-fuel and nuclear power plants are essential for maintaining grid stability and preventing blackouts, and that a rapid transition away from fossil fuels would wreak havoc on economic systems.

## Challenging the dominant narrative

For decades, proponents of climate change and sustainability were attempting to create a counter narrative but with little success. That has started to change as more and more countries enact policies to address climate change. These counter narratives recognize that there will be big costs involved in transitioning away from fossil fuels, but are also discussing the many co-benefits that can be achieved through a shift in economic growth strategies and the energy structures behind them. The environmental and climate counter narratives are offering stronger visions of what sustainable development can lead to: a brighter, renewable, clean-energy future; green and more livable urban environments; and biodiversity protection for the planet’s and human well-being.

In Germany, the sustainability narrative has arguable become the dominant narrative and now drives governmental policymaking. This is important not only for Germany but also for Europe as Germany is the largest economy within the European Union with an export-oriented manufacturing sector. After the Fukushima nuclear accident, Germany made a renewed decision to phase-out nuclear energy while phasing in renewable energy. The last of the country’s nuclear power plants is to be shut down in 2022, ending a more than 80-year history of nuclear energy in the country. In the meantime, renewable energy has expanded dramatically, now accounting for around 40% of electricity generation. Likely to miss its 2020 target for greenhouse gas emission reductions, new targets have been set for the years ahead: 55% reduction by 2030 and climate neutrality by 2050. To achieve this, the country aims at further expansion of renewables, strong energy efficiency improvements, reforestation and afforestation projects, clean technology development, hydrogen fuel and electric mobility, improved battery storage technologies, the retrofitting of buildings, resource efficiency, recycling and reuse, and changes to agricultural policy. The concepts of ecological modernization and the sustainable development goals are strongly engrained in the country. Efforts to transition the economy in more ecologically sound directions have been on-going for decades.

Various mechanisms are being introduced to enhance stakeholder involvement in implementation decisions as well as to coordinate actions across stakeholders and regions. Efforts to introduce more reflective governance structures have been made. A stakeholder committee was set up to advise the government on how best to phase out of coal in a socially just manner leading to a plan to shut down the last coal fired power plants by around 2038. Commonly known as the Coal Commission, the committee’s official name, “the Commission for Growth, Structural Change, and Employment”, speaks for itself about the importance placed not only on the main goal of the committee—to phase out coal for climate protection, but the need to address areas of great concern to those who will be impacted directly by the energy and economic transition. With the rise of the Alternative für Deutschland (AfD) and rumblings in the economic wing of the Christian Democratic Union (CDU) and within the Christian Social Union (CSU), those supporting a rapid energy transition are beginning to recognize the need to more strongly link environmental and climate protection to questions of social and environmental justice both within and between generations. With the rise of populism, this tendency has intensified as environmentalists are beginning to recognize that their environmental and climate policies are only likely to succeed if they are perceived as socially just.

## Populist and post-truth narratives

For those arguing that Germany is going it alone, the populist messages coming from the United States and several other parts of the world fuel their fears. For several decades now, there has been a disturbing trend where the United States is failing to participate in a wide range of multilateral environmental agreements. The United States has signed but not ratified a number of major international environmental agreements, including the Kyoto Protocol, and it is Donald Trump’s plan to withdraw the United States from the Paris Agreement. Behind this U.S. unilateralism is a narrative that places national sovereignty and economic choices above the longer-term benefits that can be achieved through international cooperation and collective action. A similarly disturbing development is the British choice to leave the European Union, commonly known as Brexit. Brexit was powered by a narrative that viewed European Union policies, including many of its environmental policies, as too restrictive on Britain’s economic development.

In the United States, the Tea Party movement within the Republican Party displays an antipathy towards the big government that is associated with environmentalism and the energy transition. The movement has backed the Trump administration’s decision to roll back environmental regulations meant to protect human and ecological health.

A particularly brash form of anti-environmentalism can be found among climate change skeptics and deniers. They have sought to raise doubts about climate science, to block or slow climate action, to limit government interventions into the economy, and protect the status quo. Donald Trump embraced the messages of this movement, pulled the United States out of the Paris Agreement, and rolled back many of the climate initiatives of his predecessor, Barack Obama.

Climate skepticism has spread to other parts of the world where populist political leaders have sought to remove environmental and climate restrictions in order to pursue economic growth. In Brazil, the election of populist president, Jair Bolsonaro, resulted in an opening of the Amazon to developers who burned huge tracks of rainforest to clear the land for agriculture and other purposes. Only under massive international pressure did the Bolsonaro regime take some measures to control the fires and enforcement appears lax. In numerous countries where civil society activism is feared, new restrictions have been placed on non-profit organizations’ ability to form and the activities they may partake in.

Behind these movements are powerful financial supporters, like the Koch brothers who have financed the campaigns of conservative politicians willing to support the fossil fuel industry; fossil fuel companies, like Exxon, which have supported climate skeptic science; and conservative think tanks, like the American Enterprise Institute, the Cato Institute, and the Heritage Foundation, which have sought to spread messages of doubt about climate change and environmentalism often making use of pseudo-science.

## The spread of the energy transition narrative

Germany certainly played a very important role in spreading awareness internationally in the potential of renewable energy so much so that in the meantime, Germany is no longer the world leader in installed renewable energy capacity. REN 21’s global renewable status report (REN21 2019) listed the following as the country’s with the world’s largest share of installed renewable energy capacity at the end of 2018:

- China: 727 GW (of which 322 GW hydro, 176 GW solar, 210 GW wind, and 17.8 bio)
- EU: 469 GW (of which 339 GW is non-hydro)
- India: 124 GW (of which 35 GW wind, 33 GW solar, 45 GW hydro, and 10.2 GW bio)
- Germany: 119 GW (of which 59 GW wind, 45 GW solar, 8.4 GW bio, 5.6 GW hydro)
- Japan: 86 GW (of which 56 GW solar, 22 GW hydro, 4 GW bio, and 3.7 GW wind)
- UK: 44 GW (of which 21 GW wind, 13 GW solar, 7.7 GW bio, and 1.9 GW hydro power).

In more and more countries, energy transition targets are being set and related policies being implemented. A quick survey of various developments suggests that Germany is by no means going it alone, nor is it always in the lead, but the extent to which the clean energy transition narratives have taken hold in policies varies.

## Energy transitions at the sub-national level in the United States

Many similar ideas have been embraced at the state level in the United States. California’s former Governor Jerry Brown issued an Executive Order B‑55-18 for the state to become carbon neutral and Senate Bill 100 targeting 100% carbon-emission free electricity by 2045. California has led with its emission trading system, policies to promote fuel efficiency improvements, the transition to electric mobility, and the clean tech sector. New York and a number of other states have similarly ambitious plans.

At the federal level, during the Obama administration, there was talk of initiating an Economic Recovery Plan that hinted at the idea of a Green New Deal. In his weekly television address to the nation on November 28, 2008, Barack Obama called for a “two-year, nationwide effort to jumpstart job creation in America and lay the foundation for a strong and growing economy. We’ll put people back to work rebuilding our crumbling roads and bridges, modernizing schools that are failing our children, and building wind farms and solar panels, fuel-efficient cars and the alternative energy technologies that can free us from our dependence on foreign oil and keep our economy competitive in the years ahead” (Obama 2008). In 2018, Congresswoman Alexandria Ocasio-Cortez and Senator Ed Markey respectively launched Green New Deal Resolutions in the U.S. House of Representatives and the Senate calling for radical cuts in carbon emissions to achieve carbon neutrality “through a fair and just transition for all communities and workers” while creating millions of good, high-wage jobs and investing in the country’s infrastructure and industry. The Resolution submitted to the House calls for a switch to 100% renewable energy, upgrading existing buildings in terms of their overall efficiency, and working with farmers and ranchers to eliminate pollution and greenhouse gas emissions from the agricultural sector (H. Res. 109). While their initiatives have next to no chance of gaining sufficient support to pass in Congress, they are capturing the imagination of other actors.

In January 2000, state assembly legislators in California issued their own proposal for a California Green New Deal (Assembly Bill 1839). The bill calls on California to remain a climate leader and accelerate change while highlighting the importance of assuring a just transition. It views climate policy as a chance to create new jobs but also argues that measures to assist those impacted by climate change be initiated.

Whereas the left sees the Green New Deal as a policy that will simultaneously tackle economic inequalities, promote social justice, and preserve the planet for future generations, the far right has sought to discredit the idea as ridiculously expensive, unrealistic, and communist.

Polarization has come to define U.S. politics, making policy change at the federal level difficult. It is primarily at the state level where green policy changes and energy transitions are occurring.

## The Green New Deal for Europe

The voice of citizens demanding change are influencing broader European politics as well. The European Union is actively moving to direct member states’ economies to limit resource use and waste, promote clean energy, create green jobs, redesign urban and transport structures to be more sustainable, and change societal behavior through the promotion of green consumerism. The EU is governed by dozens of framework environmental directives and hundreds of environmental regulations.

Interestingly, while the idea of the Green New Deal originated in the United States (there are various individuals claiming to be early advocates of the idea including Thomas Friedman (2019)), it has been most successful in Europe. The European Union under Commissioner Ursula Von der Leyen has issued a European Green Deal (European Commission 2019). The European Green Deal has as its main goals reaching climate neutrality by 2050, developing a more sustainable economic system, and improving the wellbeing of people. It addresses the areas of clean energy, sustainable industry, building renovation, sustainable mobility, food production and consumption, biodiversity protection and eliminating pollution all together. Among many other plans, industry is to be incentivized to use more recycled materials in its production processes and more public transportation offerings are to be made and mobility to be decarbonized. As material extraction for the production of goods contributes significantly to greenhouse gas emissions, biodiversity loss, and waste, and only 12% of materials used in industry today are recycled, this is another area of focus. Numerous plans are in development, including an industrial and digitalization strategy, which will also aim at furthering the shift towards sustainability. In the coming months many regulations and directives tied to the Green New Deal can be expected. While there is general consensus in the European Union on the importance of tackling climate change, there is only a weak agreement in relation to achieving carbon neutrality by mid-century. The real test of this policy will be in the coming decades as Green Deal policies become more concrete and need to be implemented.

## China: creating an ecological civilization

In China, there has been a remarkable shift in narratives pertaining to the environment. Whereas in the 1990s, the dominant narrative was that pollution was an inevitable consequence of economic development and that as a developing country, China should not be expected to reduce its greenhouse gas emissions, since the 2010s, the narrative has changed. President Xi Jinping has prioritized the improvement of ecological conditions, green development and reduction of environmental pollution. These are seen as critical to achieving China’s goal of becoming an “all round well-off society” (*Xiaokang society*) by 2020, achieving a basic modern economy and ecological civilization by 2035, and obtaining the status of a wealthy and “Beautiful PRC” by mid-century. Xie Zhenhua, formerly vice-director of the National Development and Reform Commission in China stated at a Brussels-based meeting of the China Council on International Cooperation on Environment and Development:

*Previously, our idea was that man can overcome nature …This kind of thinking left us with a lot of negative legacies. So we have now replaced that with the concept of ecological civilization where man exists in harmony with nature* …

*So we have clearly concluded that we have to change our development model to a green, circular, and low carbon model. Only by a transformation can we reach the goals, which we’ve set for ourselves, and achieve synergies and multi win-solutions.*^1^

The idea of an ‘ecological civilization’ appeared first around 2007 in a report to the 17th National People’s Congress (Chun 2015). In 2013 Chinese President Xi Jinpeng stated that the environment should not be sacrificed for temporary economic growth. He pledged to establish an ecological “red line” requiring all levels of government to assure that industrial development occurs within the constraints of natural conditions. “We have to understand that to protect the environment is to preserve our productivity and to improve the environment is to develop our productivity. Such concepts should be deeply rooted” (“President Xi pledges not to sacrifice environment,” *China Daily*, May 24, 2013). Arthur Hanson (2019) describes the concept of ‘ecological civilization’ as a “a philosophy, vision, and compass for a green and prosperous future” that was enshrined in the Constitution of the People’s Republic of China in 2018 and has its roots in the 12th and 13th Five Year Plans. Expectations are that the 14th Five Year Plan will further the promotion of renewable energies, energy efficiency, green consumerism, a transition to low-carbon mobility, healthy and safe food, and a circular economy. Indeed, such a transition will be essential if China is to deal with the environmental degradation caused by its increasingly well-off population with a middle class of about 400 million (Cyrill 2019). On March 11, 2020 the powerful National Development and Reform Commission and the Ministry of Justice released “Opinions Concerning Accelerating the Establishment of Regulation and Policy System on Green Production and Consumption,” which is to lead to new regulations by 2025. These include plans for ramping up cleaner energy production, smart grids, energy storage technology, and distributed energy as well as hydrogen fuel and marine energy. In addition, the opinion discusses whole-cycle and whole-chain cleaner coal exploitation and utilization.

With a population of 1.4 billion, China has a herculean challenge in trying to go low carbon. Until the past decade or so, China’s per capita emissions were still very low, but they have been growing rapidly as the Chinese economy has boomed (growing at an annual rate of around 10% per annum for the past 30 years) and the population has grown. Per capita emissions today are at about the average level of the European Union. China has fueled its growing economy largely with coal.

In recent years, China’s political leadership has focused increasing attention on restructuring its energy industry. Old and inefficient coal mines and coal-fired power plants are being shut down and renewable energy, including wind, solar, biomass, and hydro are being rapidly expanded. At the same time, however, China is building nuclear power facilities and coal fired power plants. And while renewable energy is being promoted, with major plans for further expansion, China is also investing in offshore and overseas oil exploration. Thus, in China the clean energy narrative is gaining in strength but the reliance on fossil fuels continues to be strong.

## Japan and a post-nuclear disaster, clean tech transition

After experiencing severe pollution in the 1960s and early 1970s, including deaths by Minamata mercury poisoning linked to chemical processes used in factories in the fishing town of Minimata and along the Agano River in Niigata; cadmium poisoning (itai-itai disease) linked to mining operations in Toyama; and severe respiratory problems linked to air pollution in Yokkaichi, Japan slowly changed course and became a leader in industrial pollution control, waste recycling, and the circular economy concept. The Fukushima nuclear disaster in 2011 awakened national concern about the safety of the country’s nuclear energy policies and forced the government to drastically curtail reliance on nuclear energy. Of the 54 operable nuclear reactors in 2011, 20 were permanently shut down after Fukushima. In the meantime, only a small number of the country’s 34 remaining operable nuclear reactors had been restarted (9) as of the end of 2018, and nuclear energy accounted for only about 6% of electricity (less than in Germany), down from about one-third at the time of the nuclear accident.

Although the government still aims to restart more reactors, due to public opposition and financial considerations on the part of operators, it is doubtful it will be able to achieve a 20% nuclear share by 2030 that it hopes for. The abrupt drop in nuclear capacity resulted in numerous changes in energy policies in Japan, leading to substantial gains in energy efficiency and reductions in energy demand, the initiation of support programs for renewable energy, and a greater focus on clean technology innovations. At the same time, however, Japan has resorted to expanded coal imports worsening its carbon dioxide balance.

At the local level, change is visible as well, with many local communities pursuing efforts to become nuclear-free and to develop renewable energy. Fukushima Prefecture, site of the nuclear accident, has plans to become a 100% renewable energy prefecture and a leader in new energy technologies, including hydrogen fuel. It has made large strides in this direction, especially in the electricity sector with the building of wind turbines and solar power facilities (Johnston 2018).

Beyond the energy sector, Japan has long been a leader in more sustainable transport, including the use of high speed rail, public transport, and hybrid automobiles. It is now investing extensively in ways to link new technologies, including big data, artificial intelligence, and robotization, to the development of smart cities and communities. Japan has also done an impressive job of reducing electricity demand in the post-Fukushima era.

## Discussion

As these examples from around the world suggest, there have been important shifts in the understanding of the relationships between energy, the economy and the environment. This is important as new narratives can alter how scientific information is perceived, the future worlds people can imagine, the behavior of individuals and groups, and the investment decisions made by industries. While the terminology differs somewhat among the cases examined here, new conceptions of the importance of redesigning our energy and economic systems to become more sustainable while promoting intra- and inter-generational justice are becoming increasingly powerful and entrenched. There are, however, distinct differences between the approaches being followed in major economies in North America, Europe, and East Asia.

Defenders of the status quo and those who favor incremental rather than deeper and more radical change are seeking to slow the transition towards greater sustainability. This is also the case in Germany. The rapid and large-scale energy transitions which are underway have stoked fears and contributed to populist politics. This matters as the pace of transition will determine how quickly the rise of greenhouse gas emissions will be checked, how much biodiversity will be lost, how much plastic is dumped into our oceans, and how livable our cities will be.

Despite the many initiatives discussed here and the new more sustainable narratives which are emerging, the pace and scale of change remains slow. All indicators are that much more must be done to change course to slow global warming. And new challenges are emerging as I write this piece. The shock of the Corona Virus on the global economy will be huge and it will certainly impact many policy decisions and growth trajectories. What this will mean for the struggle between different energy trajectories and climate change visions is an open question.

## Footnotes

1 Statement made at the CCICED Brussels Roundtable Event, June 19–20, 2019. Author’s notes.
